# Spontaneous Liver Rupture in the Setting of Autoimmune Disease and Periportal Edema

**DOI:** 10.7759/cureus.46079

**Published:** 2023-09-27

**Authors:** Brian Tan, Alex Dluzneski, James L Wilson, Derrick Huang

**Affiliations:** 1 Emergency Medicine, University of Central Florida, Ocala, USA; 2 Emergency Medicine, Lake Erie College of Osteopathic Medicine, Bradenton, USA; 3 Emergency Medicine, University of Central Florida, Orlando, USA

**Keywords:** autoimmune disease, periportal edema, hemorrhagic shock, emergency medicine, liver rupture

## Abstract

Spontaneous liver rupture is a rare and life-threatening occurrence associated with high morbidity and mortality. We report a rare case of an elderly patient with a significant history of autoimmune disease who initially presented with cholestatic symptomatology that subsequently resulted in spontaneous liver rupture and hemorrhagic shock. An initial CT scan prior to the rupture showed periportal edema. In a patient with unexplained abdominal pain and imaging findings of periportal edema, emergency providers should have a lower threshold for suspecting the development of liver rupture or other hepatic pathologies. In the case of a potential liver rupture, admission for observation and early resuscitation can prove key to successful treatment.

## Introduction

Spontaneous rupture of the liver resulting in life-threatening hemoperitoneum is a rare pathology usually associated with underlying hepatic diseases, including adenomas, malignancy, and hemangiomas [1]. Risk factors associated with spontaneous rupture of the liver include pregnancy, coagulation disturbances, connective tissue disorders, and hypereosinophilic syndrome [1,2]. Spontaneous rupture of the liver may follow a pre-acute phase associated with non-specific gastrointestinal symptoms, such as nausea, vomiting, and abdominal pain, which can extend to as long as a month prior to rupture [2]. Upon rupture, there may be tearing of Glisson's capsule associated with a sudden worsening of abdominal pain and signs of hypovolemic shock or hemodynamic instability [2,3]. Furthermore, rupture can also be associated with other complications, such as disseminated intravascular coagulation and pancreatitis, further complicating stabilization and management [4-7]. 

In the emergency department (ED), patients presenting with non-specific abdominal pain may carry a wide differential diagnosis ranging from atypical acute coronary syndrome (ACS) and inflammatory disease to aortic catastrophe and organ rupture [8]. An effective clinical examination and consideration of risk factors for life-threatening pathology are crucial in expeditiously risk-stratifying the patient and establishing an appropriate disposition. Here, we describe a case of spontaneous rupture of the liver with associated life-threatening hemoperitoneum in an elderly patient with several autoimmune comorbidities.

## Case presentation

A 72-year-old female with a history of ulcerative colitis, rheumatoid arthritis, psoriatic arthritis, and chronic obstructive pulmonary disease (on 2L home oxygen) presented to the ED with progressive epigastric and non-radiating right upper quadrant (RUQ) abdominal pain for two days. The pain worsened after meals that included cheese. She denied a history of similar episodes in the past. She denied fever, chest pain, shortness of breath, nausea, vomiting, and diarrhea. The patient was not on anticoagulation and did not have any recent medication changes.

**Figure 1:**
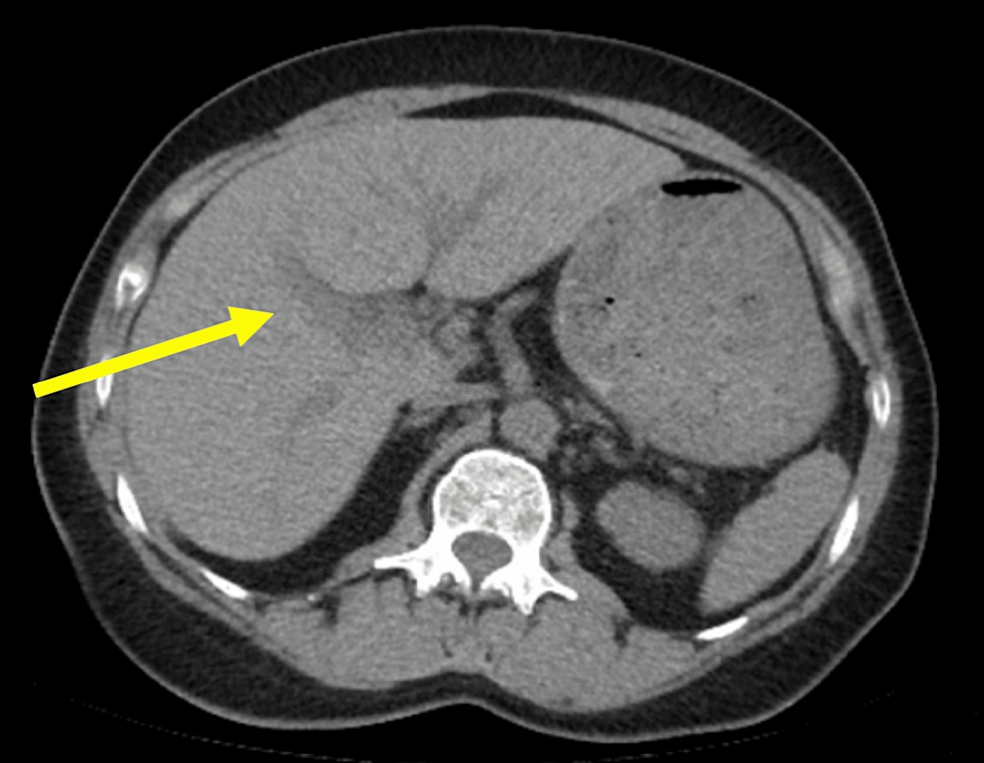
Non-contrast CT scan of the abdomen and pelvis with a transverse view showing subtle periportal edema (yellow arrow). CT: computed tomography.

On arrival, the patient was initially hemodynamically stable with vital signs in the normal range. On her initial laboratory workup, she had a WBC of 13.8x10^9^/L and hemoglobin/hematocrit of 13.4/38.6. Her initial liver function tests (LFTs) showed aspartate aminotransferase (AST) 124 IU/L, alanine aminotransferase (ALT) 85 IU/L, alkaline phosphatase (ALP) 82 IU/L, and total bilirubin 0.8 mg/dL. Her platelets, coagulation profile, lipase, and creatinine levels were unremarkable. A computed tomography (CT) scan of the abdomen and pelvis showed subtle periportal edema concerning for possible cardiogenic congestion and hepatic inflammation (Figure 1). She was subsequently admitted for persistent abdominal pain, observation, serial LFTs, and gastrointestinal consultation.

**Figure 2:**
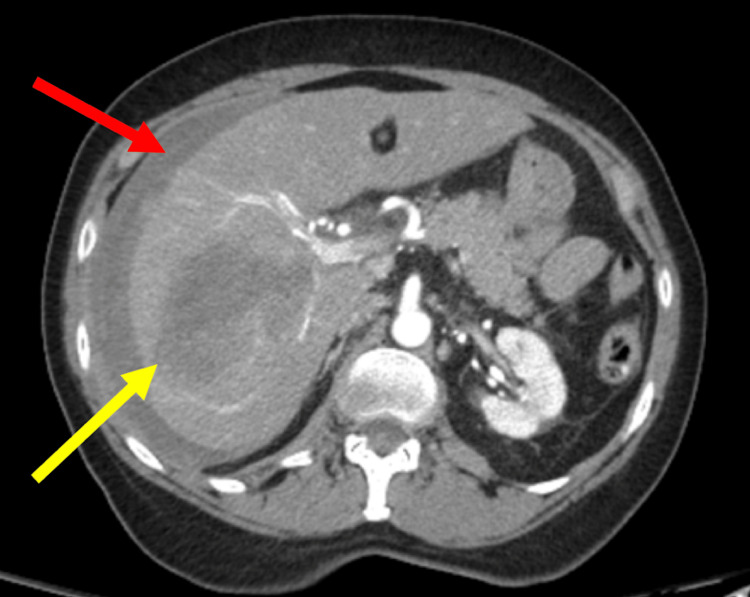
CT scan of the abdomen and pelvis with angiography with a transverse view showing a large hypodense area in the liver (yellow arrow) with disruption of the liver capsule and acute hemoperitoneum (red arrow) without active arterial contrast extravasation. CT: computed tomography.

During her admission, the patient experienced spontaneous worsening of her abdominal pain with a blood pressure of 95/52 mmHg and tachycardia. CT angiography was remarkable for acute hepatic rupture with hemoperitoneum (Figure 2). General surgery was immediately consulted, and the patient was successfully resuscitated after a prompt blood transfusion. She was then transferred to the intensive care unit. Repeat WBC and hemoglobin/hematocrit levels were 19.7x10^9^/L and 6.2/17.8, respectively. Repeat LFTs were remarkable for progressively increasing ALT, AST, ALP, total bilirubin, and direct bilirubin levels to highs of 1029 IU/L, 1500 IU/L, 365 IU/L, 8.1 mg/dL, and 4.3 mg/dL, respectively. Acetaminophen and ethanol levels were unremarkable. Although non-specific, the patient had a positive antinuclear antibody (ANA) screen, with a high ANA homogeneous pattern 1:640. Ultrasound of the RUQ, hepatobiliary iminodiacetic acid (HIDA) scan, magnetic resonance cholangiopancreatography (MRCP), and triple-phase MRI imaging were unremarkable. Although the patient recovered and was discharged without need for emergent surgical intervention per general surgery recommendations, she was readmitted soon after discharge for epigastric pain that was diagnosed as acute pancreatitis with a lipase elevation of 1604 U/L. She ultimately recovered.

## Discussion

Spontaneous liver rupture is a rare and life-threatening pathology with a diagnosis complicated by an unpredictable prodromal phase of non-specific abdominal symptoms, which has been reported to last up to one month preceding spontaneous rupture [2]. Upon rupture, the patient may experience rapidly worsening epigastric or RUQ abdominal pain associated with tearing of Glisson's capsule, shoulder pain with associated hemoperitoneum, and nausea with vomiting [2,3]. In our patient, the finding of periportal edema on CT imaging in the setting of persistent abdominal pain in a patient with multiple autoimmune comorbidities prompted admission for observation and serial LFTs, which proved prudent. After discharge, the patient was readmitted with abdominal pain that was diagnosed as pancreatitis, which may be a rare and delayed complication related to significant hepatic injury, as seen in prior case reports involving significant hepatobiliary injury [6,7]. Identification of risk factors and subtle imaging findings portending the development of life-threatening hepatic rupture was essential in determining the need for admission despite the initially benign clinical appearance of the patient and mildly elevated initial LFTs.

The diagnosis of hepatic rupture can be confirmed by ultrasound or CT scan, and further imaging studies, including angiography, triple-phase CT and MRI, MRCP, and hepatic arteriography, may help to delineate potential etiologies [4]. In our case, the initial CT scan was remarkable for periportal edema, which can be a non-specific indicator of hepatic pathology that can include congestion from heart failure, lymphatic obstruction, acute hepatitis, and traumatic causes (Figure 1) [9].

Point-of-care ultrasound (POCUS) can visualize features of both impending rupture and hemoperitoneum. For example, although ultrasound is poorly sensitive and specific, it can visualize periportal hyperechogenicity, which can be indicative of inflammatory and infectious pathologies such as viral hepatitis [10]. In the setting of hemoperitoneum, POCUS is more effective, as reflected in the established use of POCUS in the Focused Assessment with Sonography for Trauma (FAST) exam, with sensitivity and specificity values ranging from 63 to 100% and 95 to 100%, respectively [11,12]. However, a negative FAST does not exclude intra-abdominal injuries or hemoperitoneum, as retroperitoneal injuries are easily missed and the detection of hollow viscus injuries remains poorly sensitive [11,12]. Ultrasound may also assist in elucidating the etiologies of potential rupture, as pathologies such as hemangiomas can be sonographically visualized as sharp-edged hyperechoic lesions with clear borders prior to rupture [4]. In the vast majority of cases, liver biopsy is contraindicated due to an elevated risk of hemorrhage and is generally only considered in the setting of small, undifferentiated liver lesions concerning for possible hepatocellular carcinoma [4].

The liver is an integral organ in immunological defense against toxic agents and various infectious agents, leaving the liver prone to autoimmune pathology [13,14]. For example, notable autoimmune pathologies involving the hepatobiliary system include autoimmune hepatitis, primary biliary cirrhosis, and primary sclerosing cholangitis [15]. In this report, our patient had a significant history of ulcerative colitis, rheumatoid arthritis, and psoriatic arthritis, all of which are associated in varying degrees with hepatobiliary pathology ranging from portal fibrosis to hepatic and biliary cirrhosis [16-18]. Furthermore, our patient had a relatively high ANA level of 1:640 with a homogenous pattern. Although non-specific and likely related to our patient’s autoimmune comorbidities, positive ANA screening may have some utility in patients with undiagnosed autoimmune comorbidities in the setting of a possible developing hepatic rupture [19,20]. 

Conservative management is most appropriate if the patient is stable, and the liver capsule is intact [2,3]. In our case, appropriate disposition to observation and serial labs prior to hepatic rupture allowed for rapid resuscitation and recovery with conservative management. In the case of hemodynamic instability and a complete hepatic capsule rupture, hepatic artery angiographic or surgical embolization, emergency laparotomy, hepatic packing, or liver resection ranging from segmentectomy and hemihepatectomy to total hepatectomy followed by liver transplantation may be required [2,3].

## Conclusions

Spontaneous liver rupture is a rare and life-threatening occurrence that requires a high index of suspicion. The appropriate disposition of patients with the potential to experience spontaneous liver rupture may be aided by knowledge of risk factors that may include autoimmune comorbidities and subtle imaging findings that may portend an impending rupture. 
